# Prediction of in-hospital mortality after pancreatic resection in pancreatic cancer patients: A boosting approach via a population-based study using health administrative data

**DOI:** 10.1371/journal.pone.0178757

**Published:** 2017-06-07

**Authors:** Jose F. Velez-Serrano, Daniel Velez-Serrano, Valentin Hernandez-Barrera, Rodrigo Jimenez-Garcia, Ana Lopez de Andres, Pilar Carrasco Garrido, Alejandro Álvaro-Meca

**Affiliations:** 1 Department of Computer Science, Rey Juan Carlos University, Madrid, Spain; 2 Department of Statistics and Operations Research, Complutense University, Madrid, Spain; 3 Department of Preventive Medicine & Public Health, Rey Juan Carlos University, Madrid, Spain; Virginia Mason Medical Center, UNITED STATES

## Abstract

**Background:**

One reason for the aggressiveness of the pancreatic cancer is that it is diagnosed late, which often limits both the therapeutic options that are available and patient survival. The long-term survival of pancreatic cancer patients is not possible if the tumor is not resected, even among patients who receive chemotherapy in the earliest stages. The main objective of this study was to create a prediction model for in-hospital mortality after a pancreatectomy in pancreatic cancer patients.

**Methods:**

We performed a retrospective study of all pancreatic resections in pancreatic cancer patients in Spanish public hospitals (2013). Data were obtained from records in the Minimum Basic Data Set. To develop the prediction model, we used a boosting method.

**Results:**

The in-hospital mortality of pancreatic resections in pancreatic cancer patients was 8.48% in Spain. Our model showed high predictive accuracy, with an AUC of 0.91 and a Brier score of 0.09, which indicated that the probabilities were well calibrated. In addition, a sensitivity analysis of the information available prior to the surgery revealed that our model has high predictive accuracy, with an AUC of 0.802.

**Conclusions:**

In this study, we developed a nation-wide system that is capable of generating accurate and reliable predictions of in-hospital mortality after pancreatic resection in patients with pancreatic cancer. Our model could help surgeons understand the importance of the patients’ characteristics prior to surgery and the health effects that may follow resection.

## Introduction

Although the global mortality associated with cancer has decreased by approximately 10% in recent years, pancreatic cancer is an exception [1]. This reduction in mortality is attributable to advances in cancer treatment, among other reasons. Nevertheless, pancreatic cancer remains the fourth most frequent cause of tumor-related death in the western world [2]. One of the reasons for the aggressiveness of pancreatic cancer is that it is often diagnosed late [3], which frequently limits the available therapeutic options and patient survival [4].

The long-term survival of pancreatic cancer patients is not possible if the tumor is not resected, even when patients receive chemotherapy in the earliest stages [3]. Nevertheless, pancreatectomy is associated with a high in-hospital mortality rate that is affected by multiple risk factors, such as demographic characteristics, co-morbidities, hospital volume and surgeon experience [5–9]. To the best of our knowledge, no national-level study has performed a thorough analysis of the use of machine learning techniques to predict the in-hospital mortality risk for pancreatic cancer patients after pancreatectomy.

In recent years, predictive models have been developed to determine the perioperative risk of pancreactectomy [5, 10, 11]. Nonetheless, these models use very limited information to classify patients. Indeed, patient classification plays a key role in modern clinical research. The goal of binary classification schemes is to divide subjects into two mutually exclusive categories based on their observed characteristics [12]. Furthermore, data mining techniques, such as boosting [13], are very powerful tools for making predictions and classifying patients. The main objective of this study was to create a prediction model for in-hospital mortality after pancreatectomy among pancreatic cancer patients using the modern technique of machine learning, specifically a boosting method.

## Materials and methods

### Study design and data source

Data were obtained from the records of the Minimum Basic Data Set (MBDS) of the National Surveillance System for Hospital Data in Spain, provided by the Spanish Ministry of Health. The MBDS is a clinical and administrative database that contains information that is obtained and recorded at the time of hospital discharge. It has estimated coverage rates of 97.7% and 25% of total hospital admissions to public and private hospitals, respectively [14]. The MBDS provides the encrypted patient identification number; sex; date of birth; dates of hospital admission and discharge; medical institutions providing the services; the diagnosis and procedure codes according to the International Classification of Diseases, 9th ed, Clinical Modification (ICD-9-CM); and outcome at discharge (http://www.icd9data.com/2007/Volume1/140-239/default.htm).

### Pancreatic cancer cohort identification and outcomes

The primary outcome was in-hospital mortality defined as mortality during the same hospitalization as the pancreatic resection. All hospital admissions with pancreatic cancer defined at first diagnosis were selected, as defined using ICD-9-CM code 157. We selected all hospital discharges of patients older than 20 years between January 1, 2010, and December 31, 2013, who underwent a pancreatic resection defined according to ICD-9-CM codes 52.51, 52.52, 52.53, 52.59, 52.6, and 52.7. From this primary cohort, we randomly selected 75% of the sample as a training set; the remaining 25% of the sample formed the testing set.

### Predictor variables

The selected predictors was based on the demographic characteristics, hospital volume, diagnosis-related death codes and type of pancreatectomy.

- As demographic characteristics, the patient’s sex and age by decade were selected.
- Hospital volume was defined as the number of pancreatectomies performed by each hospital per year.
- As diagnosis-related death information, we selected all diagnosis codes that were related to death in our training set.
- The type of pancreatectomy was defined as the presence of any of the following ICD-9-CM codes: 52.51, 52.52, 52.53, 52.59, 52.6, and 52.7.

### Model development

For classification and prediction, we employed boosting, which is one of the most promising extensions used in data-mining and machine learning. Boosting is a method that combines “the outputs from several “weak” classifiers to produce a powerful “committee” [13]. A weak classifier is any prediction model that is slightly better than a random one.

In this work, we use the *AdaBoost.M1* model proposed by Freund and Schapire [15]. AdaBoost trains a weak classifier *c*_1_ over whole set of training samples *S* = {*s*_1_, *s*_2_…*s*_|*S*|_}. As *c*_1_ is a weak classifier, some samples os *S* will be misclassified. To improve the results, a second weak classifier *c*_2_ is trained. In this second training process, a higher weights are assigned to the samples misclassified by *c*_1_. So, *c*_2_ has been designed to get success in those samples in which *c*_1_ failed. This process is repeated, and a sequence of classifiers *C* = {*c*_1_, *c*_2_,…*c*_|*C*|_} are generated. Finally, the predictions made by this sequence of classifiers are combined into one unique prediction function *h*(*s*_*i*_, *survive*) using a weighted voting process [12, 16, 17].

Although the classifiers used as weak classifiers could be as simple as euclidean classifiers, it is common to use classification trees. It has been proven [13] that AdaBoost, even when decision trees with only one level of depth (“stumps”) are used, outperforms unique deep decision trees. Finding an optimal criterion to choose the depth of the decision trees used as weak classifiers is difficult [12]. Therefore, we tested different alternatives. Specifically, we tested trees of depth 1, 2, 3 and 4. Additionally, we tested different alternatives for the number |*C*| of classifiers in the sequence (from |*C*| = 100 to |*C*| = 5000).

We tested the predictions using three different measures. First, we calculated the receiver operating characteristic curve (ROC curve) and then calculated the area under the curve (AUC). Second, we calculated the Brier score [18], which is defined as the prediction mean square error, where low values (near zero) indicate an accurate prediction [19, 20]. Also, the Accumulated Captured Response plot is calculated (ACR).

The trained boosting classification algorithm *h* predicts if some patient *s*_*i*_ will survive after a pancreatectomy by analysing if *h*(*s*_*i*_, *survive*) > *h*(*s*_*i*_, *dead*). But the *h* function does not give the survival probability of the patient *P*(*survive*|*s*_*i*_). In several applications, correctly predicting the probabilities is important. Therefore, we applied the Platt Transform [21], in which a logistic function (1) is used to obtain this probability.

$$\begin{array}{c} P\left(survive|s_{i}\right)=\frac{1}{1+exp(A\cdot h\left(s_{i},survive\right)+B)} \end{array}$$ (1)

Where *A* and *B* are two scalar parameters estimated using a maximum likelihood method. Initially, this transform was applied to predictions obtained using Support Vector Machines (SVM) [21]. However, the application of the transformation after boosting is usual too [22].

One frequent criticism mentioned in relation to some machine learning techniques is the difficulty of explaining the importance of each variable in the adjusted model. This is why we calculated the relative importance of each variable in the classification by using the Gini index for each variable in a decision tree [23]. Also, we built a regresion tree to understand the profile of the patient to which the model assigned higher mortality. To generate this tree, we use the calculated *P*(*survive*|*s*_*i*_) as dependent variable. Then, for each input variable, we perform a Fisher test and identify the variable with the lowest p-value. Because the age and hospital volume variables are not binary, in this case we generated several two-class partitions, and we used the Fisher test to select the best one. Subsequently, we create two nodes that divide the previous samples according to the variable being analyzed. This process is repeated until no p-values less than 0.05 remain, the number of samples in a node is insufficient, or the tree becomes deeper than a fixed threshold.

Finally, we perform a sensitivity analysis of the pre-surgery in-hospital mortality predictions. This analysis was conducted by removing the variables that belong to post-surgery processes. Again, we present the Brier score, prediction calibration, ROC curve and ACR curve.

Overall, the results are presented as the mean (95% confidence interval [95% CI]) for continuous variables and as frequencies and percentages for categorical data. The trends in the categorical data were evaluated with a Mantel-Hanztel *χ*^2^ test, and p-values <0.05 were considered significant. All analyses were performed using the adabag [24] library from the R [25] platform.

### Ethical aspects

The data were treated with full confidentiality according to Spanish law. Given the anonymous and mandatory nature of the data, patients cannot be identified at the individual level in this paper or in the database; thus, informed consent for this study was not required. The Spanish Ministry of Health confirmed that our study fulfilled all ethical considerations according to Spanish law.

## Results

### Characteristics of the study population

Overall, between 2010 and 2013, 4,088 pancreatic resections were performed in Spain on patients with a primary diagnosis of pancreatic cancer. Of these, 347 (8.49%) died in hospital. Most of the patients were men (55%), and 9.4% of the men and 7.4% of women who had undergone surgery died. The average age of the patients was 64 years, and the average age of those who died was 70 years old(Table 1). Most of the patients had a primary diagnosis of a malignant neoplasm of the head of the pancreas (63%), and these patients also exhibited higher in-hospital mortality (8.6%). The most common co-morbidities among these patients were high blood pressure and metastasis (37%), followed by diabetes without complications (26%), the IHM for those suffering these comorbid conditions were 6%, 7% y 6% respectively (Table 1). Moreover, patients with congestive heart failure (2%) exhibited the highest death rate (31%) (Table 1). Most of the resections performed were partial pancreatectomies (65%), and 7.34% of the patients who underwent this procedure died. Total pancreatectomies considered as the sum of subtotal, total and pancreaduodenectomy constituted 36% of all of the pancreatectomies performed in Spain, and 11.3% of these patients died in the hospital (Table 1). Finally, hospital volume exhibited differential behavior according to the number of surgeries performed by year; thus, the hospitals that performed more than 24 surgeries per year had lower in-hospital mortality (7%) than those that performed fewer surgeries (11% in hospitals performing fewer than 13 surgeries per year vs. 8% in those performing between 13 and 24 surgeries per year; p<0.001 (Table 1).

**Table 1:** Summary of the clinical characteristics of pancreatic resection.

Description	Overall	Mortality
**No. of patients**	4088	347 (8.48)
**Males**	2244 (54.89)	211 (9.4)
**Age (years)**	63.97 (63.61; 64.32)	70.31 (69.37; 71.25)
**Length of stay (days)**	23.96 (23.38; 24.54)	31.96 (28.32; 35.6)
**In-hospital death**	347 (8.49)	347 (100)
**Diagnosis type**		
Malignant neoplasm of the head of the pancreas	2581 (63.14)	221 (8.56)
Malignant neoplasm of the body of the pancreas	242 (5.92)	13 (5.37)
Malignant neoplasm of the tail of the pancreas	288 (7.05)	7 (2.43)
Malignant neoplasm of the pancreatic duct	46 (1.13)	4 (8.7)
Malignant neoplasm of the islets of Langerhans	57 (1.39)	1 (1.75)
Malignant neoplasm of other specified sites of the pancreas	375 (9.17)	34 (9.07)
Primary malignancy of the pancreas	499 (12.21)	67 (13.43)
**Comorbid conditions**		
Hypertension	1526 (37.33)	101 (6.62)
Myocardial infarction	75 (1.83)	11 (14.67)
Congestive heart failure	83 (2.03)	26 (31.33)
Periphral vascular disease	106 (2.59)	18 (16.98)
Cerebrovascular disease	40 (0.98)	8 (20)
Dementia	7 (0.17)	0 (0)
Chronic pulmonary disease	290 (7.09)	33 (11.38)
Connective tissue disease-rheumatic disease	41 (1)	2 (4.88)
Peptic ulcer disease	65 (1.59)	10 (15.38)
Mild liver disease	322 (7.88)	58 (18.01)
Diabetes without complications	1073 (26.25)	64 (5.96)
Diabetes with complications	38 (0.93)	4 (10.53)
Paraplegia and hemiplegia	6 (0.15)	1 (16.67)
Renal disease	92 (2.25)	14 (15.22)
Moderate or severe liver disease	43 (1.05)	8 (18.6)
Metastatic carcinoma	1529 (37.4)	111 (7.26)
HIV	14 (0.34)	1 (7.14)
**Type of pancreatic resection**		
Proximal pancreatectomy	1848 (45.21)	148 (8.01)
Subtotal panceatectomy	139 (3.4)	14 (10.07)
Total pancreatectomy	624 (15.26)	88 (14.1)
Pancreaduodenectomy	724 (17.71)	67 (9.25)
Distal pancreatectomy	618 (15.12)	31 (5.02)
Other partial pancreatectomy	203 (4.97)	14 (6.9)
**Hospital surgical vol.**		
Low (<13/yr)	1010 (24.71)	117 (11.58)
Medium (13-24/yr)	1169 (28.6)	95 (8.13)
High (>24/yr)	1909 (46.7)	135 (7.07)

### Predictive accuracy, calibration and variable importance

**Fig 1:**
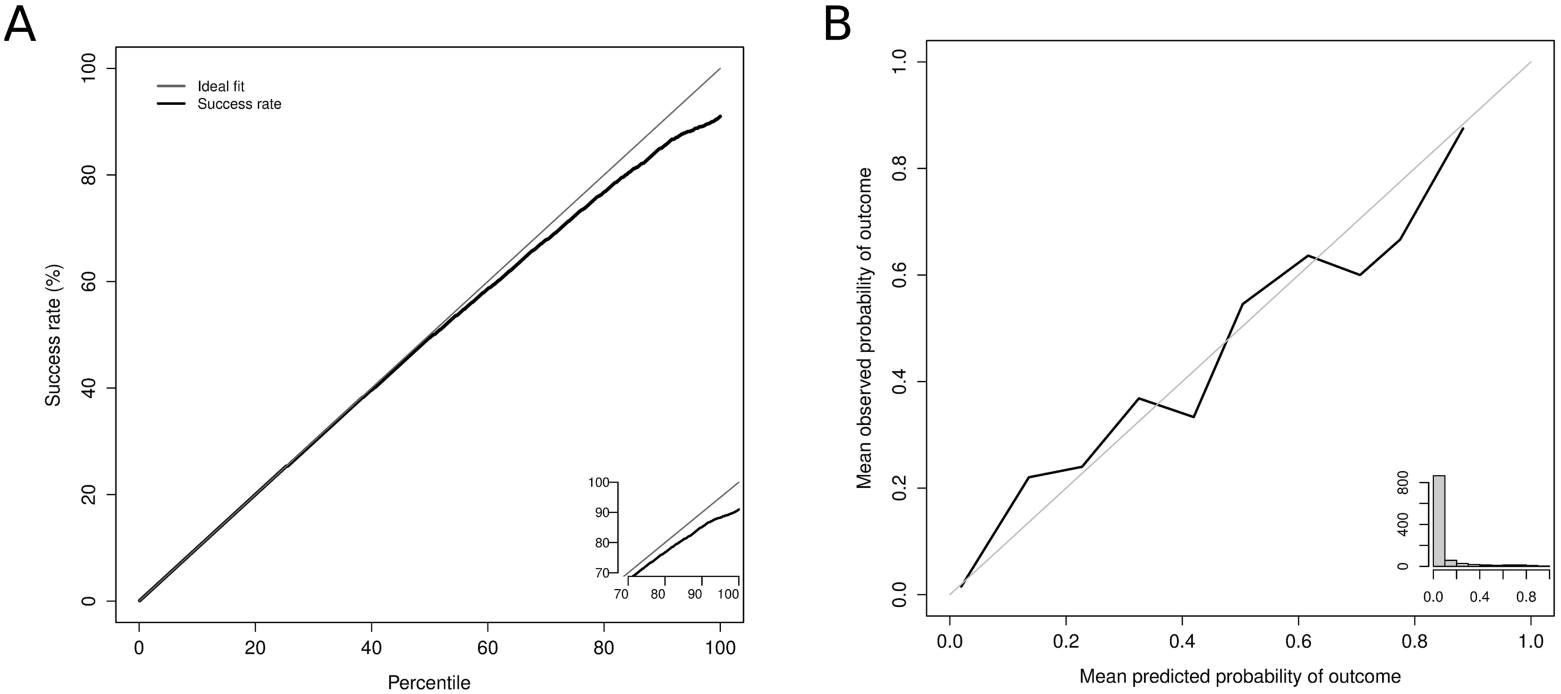
Cumulative success rate in test sample **(a)**. Calibration of the prediction of in-hospital mortality in the test sample **(b)**.

**Fig 2:**
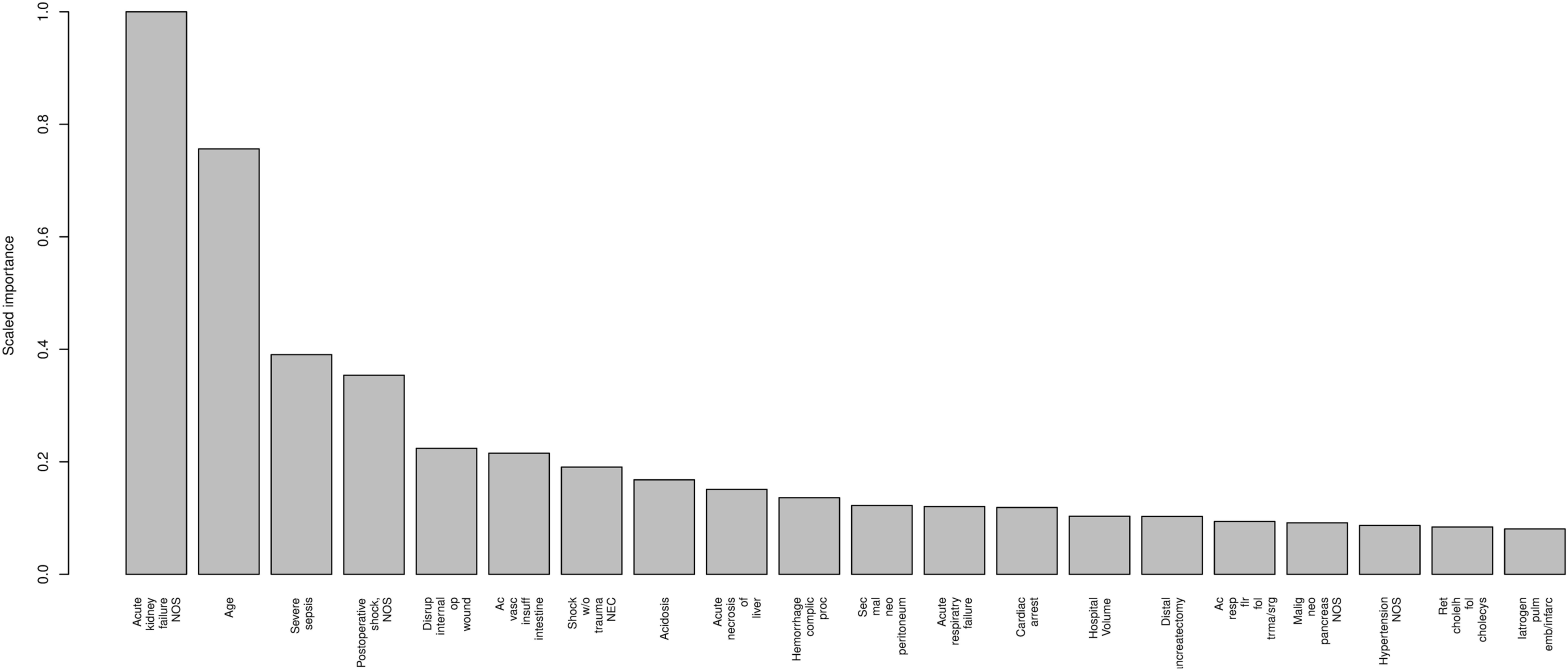
The importance of each variable. Each bar represents the gain in the Gini index attributable to each variable used to boost the weight of the tree. Only the first 20 variables are plotted.

**Fig 3:**
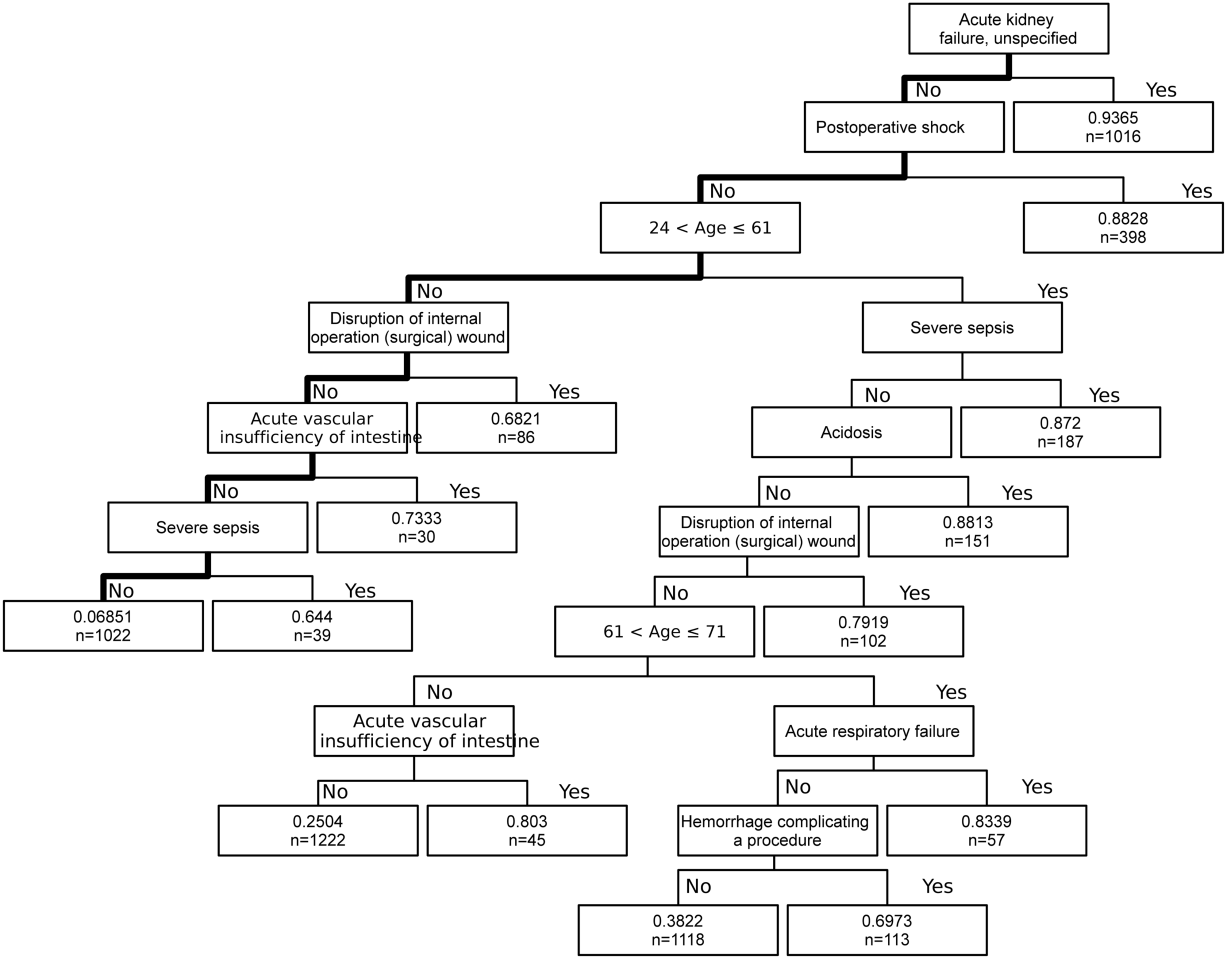
Regression tree of the predicted probabilities using variable importance.

In our model, we considered the 564 variables that were related to death in the training set. Based on these variables, we built several architectures (i.e., by varying the depth of the classification tree from 1 to 4). Finally, we chose a 3-depth model that allowed us to study interactions with a 1980-classification tree sequence. This model was chosen because it achieved the best balance among the AUC, Brier score and calibration (data not shown). The final architecture showed a high predictive accuracy in the validation set for in-hospital mortality after pancreatic resection with an AUC of 0.916 and a Brier score of 0.09. In many cases, obtaining a large area under the curve is not sufficient; indeed, it is more important to achieve a good calibration of the probabilities throughout the range of predictions. Fig 1a presents the accumulated captured response plot. This figure shows that up to the second quartile, the system’s predictions do not fail; furthermore, the error does not reach 2% until above the third quartile. Fig 1b shows the calibration of the obtained probabilities. The probabilities are clearly accurate, and thus, our results coincide quite well with the ideal fit. In our analysis of the importance of the variables, 134 of the 564 variables that were involved in the model’s construction are the most strongly related to the prediction’s result. As can be seen in Fig 2, the variables more used by the weak learners are: acute kidney failure, age, severe sepsis and postoperative shock. The regression tree obtained is shown in Fig 3. It can be observed that the variables positioned in the first nodes of the tree coincide with the variables more used by the weak learners.

### Sensitivity analysis

**Fig 4:**
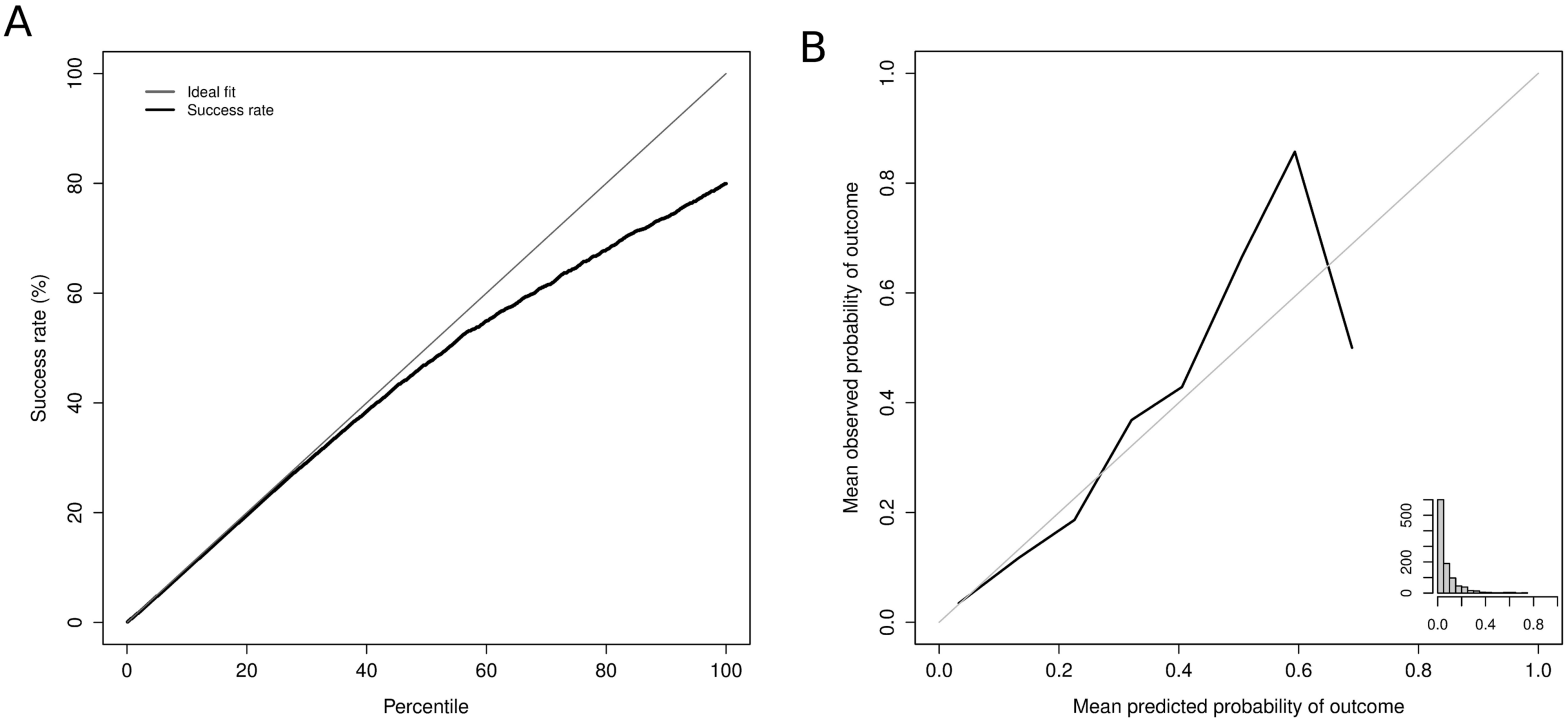
Cumulative success rate in the sensitivity test sample **(a)**. Calibration of the prediction of in-hospital mortality in the sensitivity test sample **(b)**.

The sensitivity analysis verified the high predictive capacity for pre-surgery in-hospital mortality, with an AUC of 0.802 and a Brier score of 0.169. Fig 4a presents the accumulated captured response plot. This figure shows that up to the second quartile, the system’s predictions do not fail; furthermore, the error reaches 20% only above the third quartile. Fig 4b shows the calibration of the probabilities, demonstrating poorer behavior than when all the information on the hospital stay is used (Fig 1b). Indeed, the system underestimates the death rates and deviates from the ideal fit Fig 4b. When we analyze the preoperative variables associated to in hospital mortality we identified that the most strongly related were age, Diabetes mellitus without mention of complication, and Hypertension. A quality improvement program should improve the control of diabetes and hypertension prior to surgery as this may have an impact in the short term results of the surgery. However further investigations are required to verify this hypotesis.

## Discussion

In this study, we developed a model that can classify and make accurate and reliable predictions for in-hospital mortality among patients with pancreatic cancer who undergo pancreatic resection. Additionally, we demonstrated that the use of pancreatic resection in pancreatic cancer treatment is associated with high postoperative mortality (the in-hospital mortality in Spain was determined to be 8.48%). This mortality rate is affected by the hospital volume.

Our results revealed a global mortality rate of 8.48%. This value is surprisingly high compared to those of previous reports [5, 6, 9, 26, 27] but is aligned with some other reports publishing mortality rates close to 10% [28–30] and up to 14% [31]. These differences may be attributable to the fact that the studies reporting lower rates were conducted in individual centers [9] with high volumes of surgeries and experienced surgeons; indeed, these variables have been proven to enhance in-hospital mortality. Nevertheless, one recent systematic review [32] revealed that the in-hospital mortality after pancreatic resection is approximately 6%, which is significantly lower than that observed in Spain.

Very recently Nimptsch U et al [33] analyzed all inpatient (58,003) with a pancreatic surgery procedure code in Germany from 2009 to 2013 using nationwide administrative hospital data. The results showed that the overall in-hospital mortality rate was 10.1% and did not significantly change during the study period. Major pancreatic resections were associated with mortality ranging from 7.3% (distal pancreatectomy) to 22.9% (total pancreatectomy). In the US using Texas Medicare data (2000–2012), Mehta et al [34] reported a 9% 30-days mortality, very close to our result.

Wilde et al [35], in an study performed in the Netherlands between 2004 and 2009, showed that the in hospital mortality after pancreaticoduodenectomy was 14.7%, 9.8, 6.3 and 3.3 per cent in very low, low-medium and high-volume hospitals respectively. The mortality rate after pancreaticoduodenectomy in patients >70 years was 10.4% compared with 4.4% those under this age. These authors used a database similar to ours with mortality rates close to those found in our investigation.

The variables identified as the most important in the present study are in agreement with some previous works [5–8]. Here, age and hospital volume, as reported elsewhere, were shown to be relatively important [5–8]. Our method allowed us to detect other influential preoperative and postoperative variables. Acute liver necrosis was identified as being quite relevant in our model, as were the presence of different types of secondary neoplastic malignancies and prior myocardial infarction. The variables with postoperative importance were identified as follows: acute kidney failure, sepsis, cardiac arrest and postoperative shock. The model developed here allowed us to study pre- and postoperative conditions rather than focusing on only preoperative [5] or postoperative conditions [11].

Certainly the percentage of metastasis collected in the MBDS is high, perhaps this may be due to an overcoding problem, however in the work of Grendar et al [36] using the Healthcare Cost and Utilization Project Nationwide Inpatient Sample database, they found that In patients who underwent pancreatic resection, 25.5% had metastasis, although there is still a large difference with our series. An explanation for this fact, could be the difference in the type of patient, in Spain the patients have a high mean age, long hospital stays and a high prevalence of comorbidities [36]. It is also important to point out that in Spain the health system is public, universal and free and is not governed by principles of efficiency. This is why patients with metastasis may undergo surgery as a palliative treatment. This is coherent with the results for IHM that is lower among those with metastasis (7.26%) than for those without (9.08%).

Typically, logistic regressions have been used to create predictive models for this pathology and its treatment [5, 10, 11]. However, the restrictions on linearity, variable collinearity and number of variables to introduce of linear regressions are well known. Thus, in this study, the boosting method, a machine learning technique, was used to build a predictive model for in-hospital mortality after pancreatic resection. This technique allowed us to overcome the restrictions of logistic regression and thus widen the framework of the problem to include hundreds of variables. Boosting made including the individual information of each patient’s diagnosis, thereby avoiding the aggregation of diagnoses as in typical co-morbidity indexes, possible [37, 38]. We believe that this method allowed us to achieve an AUC of 0.916 in the validation set, which is considerably higher than those of other approximations, which generated values of barely 0.72 [5, 6, 10, 11]. Moreover, the proposed model also achieved success rates exceeding 90% and well-calibrated probabilities. Even when only the patient information available prior to surgery was considered, we obtained AUCs of 0.802, thus confirming that this model’s classifying power is higher than those of previously used methods.

To the best of our knowledge, this is the first study in which all diagnoses related to death were used to build a predictive model instead of the co-morbidity indexes [5, 6] or the co-morbidities that compose such indexes [39]. This is mainly attributable to two reasons: First, the use of a co-morbidity index does not provide a differential element when classifying patients because even when patients exhibited the same values (for example, the same demographic characteristics, same hospital volume and the same co-morbidity index), some dies and others did not. Thus, making a good classification was difficult. Nevertheless, it must be acknowledged that these indexes have been demonstrated to exhibit a powerful relationship with in-hospital mortality after pancreatic resection [7, 40]. Second, among the co-morbidities that compose these indexes, co-morbidities as different as, for example, diabetes without complications and uncontrolled type II diabetes with coma are supposed to have equal contributions because quantifying their relative weights is impossible.

The present study has several limitations. First, our conclusions are limited to the application of the boosting method to classifying and predicting in-hospital mortality among patients diagnosed with pancreatic cancer and having undergone pancreatic resection. Our results cannot be generalized to the out-of-hospital mortality. Furthermore, these results cannot be extended illnesses or procedures other than those discussed here. Second, limited research on the optimal tree depth has been conducted. We tested different depths, but this does not mean that, using this architecture, the optimal or most accurate results will always be achieved. Third, the MBDS is a powerful tool for studying and understanding outcomes after surgery at the national level. Nevertheless, the restrictions inherent in the use of administrative databases must be considered. The data were obtained using ICD-9-CM for malignant neoplasms and pancreatic resection. Thus, the diagnoses of the patients who died may be subject to over-codification. Furthermore, the MBDS does not collect relevant clinical information about preoperative conditions, such as the stage of tumor metastasis, chemotherapy, radiotherapy, and lifestyle, which could contribute to improving the predictions.

Unfortunately with the MBDS database is not possible to know if these patients were found to have metastasis at laparotomy and if they had their resection aborted or if there was a curative intent surgery. Furthermore, we don’t know if the metastatic disease was identified preoperatively or intraoperatively. We agree that future investigation could be conducted in patients with curative intent surgery.

## Conclusion

In summary, in this study, we developed a nation-wide system for accurately and reliably predicting in-hospital mortality after pancreatic resection in patients with pancreatic cancer. Our model could help surgeons understand the importance of the characteristics of patients prior to surgery and the health effects that may follow pancreatic resection. A direct application of our investigation in order to reduce in hospital mortality would be a better control prior to surgery of those modifiable risk factors that increase mortality.
